# The acute effects of working time patterns on fatigue and sleep quality using daily measurements of 6195 observations among 223 shift workers

**DOI:** 10.5271/sjweh.3964

**Published:** 2021-08-31

**Authors:** Hardy A van de Ven, Gerben Hulsegge, Thijmen Zoomer, Elsbeth M de Korte, Alex Burdorf, Karen M Oude Hengel

**Affiliations:** Netherlands Organisation for Applied Scientific Research TNO, Department of Sustainable Productivity and Employability, Leiden, The Netherlands; Netherlands Organisation for Applied Scientific Research TNO, Department of Work Health Technology, Leiden, The Netherlands; Erasmus University Medical Center, Department of Public Health, Rotterdam, The Netherlands

**Keywords:** Key terms backward rotation, ecological momentary assessment survey, forward rotation, night shift, quick return, shift work, work schedule tolerance

## Abstract

**Objectives:**

This study aimed to estimate acute effects of roster characteristics on fatigue and sleep quality and investigated whether these effects differed by individual characteristics.

**Methods:**

Using an ecological measurement assessment survey, fatigue and sleep quality were daily measured among 223 shift workers for up to eight weeks. A questionnaire assessed baseline characteristics, and roster data were retrieved from the company registers to determine roster parameters. The effects between each shift parameter on fatigue and sleep quality were estimated with random- and fixed-effects models.

**Results:**

Compared to day shifts, night shifts were related to fatigue [β=0.22; 95% confidence interval (CI) 0.05–0.39] and poorer sleep quality (β =0.64; 95% CI 0.47–0.80), and more successive night shifts with more fatigue (up to β=0.68; 95% CI 0.49–0.87 for ≥2 nights). Fatigue was increased after a quick return (<11 hours) (β=1.94; 95% CI 1.57–2.31) or 11–16 hours (β=0.43; 95% CI 0.26–0.61) compared to >16 hours between shifts. Compared to forward rotation, stable (β=0.22; 95% CI 0.01–0.43) and backward rotation (β=0.49; 95% CI 0.23–0.74) were also associated with more fatigue. Workers with a morning or intermediate chronotype had poorer sleep quality after a night shift, while workers with poor health reported poor sleep quality as well as more fatigue after a night shift.

**Conclusions:**

To alleviate acute effects of shift work on fatigue, shift schedules should be optimized by ensuring more time to recover and rotate forwards.

To serve the economic and societal demands of the 24/7 economy, shift work has increased with more and more people working outside a traditional and consistent daytime schedule. Shift work that includes night work is a highly prevalent occupational risk factor. About 15–20% of the workforce in Europe and the US works in irregular shifts (1). Engaging in shift work – particularly in night shifts – has a direct negative effect on the productivity of workers (2, 3), while it has also been linked to severe health problems, such as diabetes mellitus type 2 (4), cardiovascular disease (5), and all-cause mortality (6). In addition to work-induced fatigue due to mentally or physically demanding tasks, shift work disrupts the normal sleep–wake cycle leading to sleep problems. When there is insufficient time to recover, sleep problems and fatigue may accumulate and result in chronic fatigue (7–10). Disturbed sleep and fatigue lead to dysregulation of many bodily functions, such as glucose metabolism, the excretion of hormones and the functioning of the autonomic nervous system (11–15), which increase the likelihood to develop chronic diseases in the long term (4, 5, 10).

The design of a shift system is one of the key modifiable factors that affects sleep and fatigue (16–18). Specific roster characteristics such as successive (night) shifts, backward rotation (ie, counterclockwise start times of successive shifts) and quick return to work (ie, limited time between shifts) have been associated with sleep and fatigue (19–21). However, knowledge to optimize the best configuration of a shift system is lacking (18). The absence of reliable knowledge on the effects of different roster characteristics could be explained by the fact that most previous studies compared shift workers with non-shift workers, included only shift workers in traditional collective (regular) shift systems in which all workers have the same type of roster, and used retrospective surveys to assess exposure (ie, shift schedules) and sleep parameters. Furthermore the work setting may also matter. Many studies on rosters, sleep and fatigue have been conducted in healthcare, industry and offshore, whereas studies in the entertainment industry are scarce. Additionally, the effects of different rosters may also depend on individual differences, which have rarely been investigated in longitudinal studies (18). When shift work is inevitable, insight is needed into specific roster characteristics that are related to sleep and fatigue, and differences between individuals, in order to find optimal shift schedules to minimize night-work related health problems.

Register-based assessment of working times is a reliable method to analyze large quantities of individual shift schedules over a long period of time (22). So far, register data on working time patterns have been linked with retrospective surveys to examine medium to long term effects of roster on sleep and fatigue (20, 23) or focus on the sleepiness dimension of fatigue rather than the exhaustion dimension (lack of energy). Although sleepiness and lack of exhaustion correlate well, exhaustion also accumulates over a shift cycle (24). Daily information of both roster characteristics and outcomes over a prolonged period provide insight into the dynamic changes in workers’ patterns regarding shift work and outcomes. Garde et al (25) investigated the impact of three predefined shift work schedules on sleep quality and duration among police officers by using daily time measurements in a cross-over design. They found that sleep duration was reduced after night shift work and was not further affected with more successive night shifts, while sleep quality was the poorest after the last night shift. By using daily measurements of workers with shift work schedules that differ between and within individuals, it is also possible to include other roster characteristics such as quick returns to work and rotation direction of shifts. Additionally, daily measurements will improve exposure assessment and reduce misclassification in comparison to common longitudinal studies with only a few measurements of risk factors and outcome of interest. Furthermore, it is possible to investigate differences across individuals using in a random-effects model for example, but also to investigate changes within individuals, using a fixed-effects model. Within a fixed-effects model, each individual functions as his or her own control, which removes time-invariant confounding, and improves casual inference (26).

The objectives of the current study were to estimate acute effects of roster characteristics on the exhaustion dimension of fatigue and sleep quality in the entertainment industry, and to investigate whether these effects differed by individual characteristics (ie, self-perceived health and chronotype) by using daily measurements over a period of eight weeks among shift workers.

## Methods

### Study population and study design

A field study was conducted among workers in rotating shifts at 14 worksites of an entertainment company in The Netherlands, comprising five departments providing (1,2) two sorts of entertainment, (3) preparing and serving food and beverages and (4) front desk services and (5) security. All work tasks were predominantly physically demanding (eg, standing most of the time), included interaction with visitors and were for those providing entertainment in particular also cognitive-demanding (concentration). Most of the workers participated in self-rostering, and some had a predetermined roster. Companies were open to customers 12:00–04:00 hours, except for two worksites that operate 24/7.

Data collection consisted of a baseline questionnaire, an ecological momentary assessment (EMA) survey application and roster data. First, a baseline questionnaire was digitally distributed to collect individual characteristics of the workers including sociodemographics, chronotype, and health status. Second, a smartphone-based EMA survey application was used to collect daily data over a period of eight weeks about fatigue and sleep quality. Third, the EMA data was linked with roster data to determine the roster characteristics. Rosters were received from the human resource (HR) department of the company with data on the daily start and end times that workers had actually worked. All roster characteristics were linked to individual baseline data and the daily data on fatigue and sleep quality.

The company had 3366 workers who work in shifts. We invited 869 workers who completed the baseline questionnaire to participate in the daily EMA survey measurements, of which 305 (35%) participated at least one day. Workers with data on sleep and fatigue for <7 days (N=70) or with missing data on any of the covariates (N=12) were excluded. Finally, 223 workers were included with a total of 6195 observations. The Netherlands Organisation for Applied Scientific Research’s review board approved this study (TNO-2019-084). This internal ethics committee declared that the Medical Research Involving Human Subjects Act does not apply. All participants digitally signed the informed consent form.

### Fatigue and sleep quality

Workers were instructed to complete the questions on fatigue and sleep quality with the EMA app right after their shifts on workdays, and at the end of the day on days off. Fatigue was measured daily with the question: “How would you describe your level of fatigue during the day?”. This question was based on the validated single-item fatigue scale (27), reflecting mostly exhaustion after a day’s work and to a lesser extent current sleepiness. Workers could answer these questions on a scale ranging from 0–100, with 0=“not at all tired” and 100=“extremely tired”.

Sleep quality was assessed daily with the question: “How well have you slept during your last long sleeping period?”, based on the validated single-item sleep quality scale (28). This question was paired with the instruction: “Think about how easily you fell asleep, how many times you woke up during sleep and how well rested you were after you woke up.” Workers could answer these questions on a scale ranging from 0=“very bad” to 100=“very good”.

Fatigue and sleep quality were rescaled to scores ranging from 0–10, with higher scores indicating more fatigue and poorer sleep quality so outcomes could be assessed uniformly.

### Roster characteristics

Based on the roster data of the actually worked shifts, the following shift work parameters were determined: type of shift, rotation direction and time between shifts.

Type of shift was categorized into six different shifts: (i) a day off following a workday; (ii) ≥2 days off after a working day; (iii) morning shift (starts after 06:00, before 11:00 and mostly at 07:00 hours); (iv) day shift (starts after 11:00 and before 14:00 hours); (v) evening shift (starts after 14:00 and ends before 01:00 hours); (vi) night shift (starts after 14:00 and ends after 01:00 hours). Shift length was usually ≤9 hours including breaks. Based on the type of shift, the number of successive night shifts were calculated and categorized into one, two, three and four or more successive nights shifts.

Rotation direction was categorized into (i) forward (change to a later shift), (ii) stable (the same type of shift in a row) and (iii) backward (change to an earlier shift). If there were >2 successive days off, the chain of shifts is broken, and the rotation direction resets.

Time between shifts was categorized into (i) <11 (quick return); (ii) 11–16; and (iii) >16 hours of rest between shifts.

The reference category for all roster parameters is set to the expected best option according to existing recommendations for shift schedules (16, 18). Day shift, ≥16 hours rest between shifts, forward rotation, and no successive night shifts were set as reference.

### Covariates

All covariates were assessed with the baseline questionnaire. Age, gender, education, having children aged ≤12 years living at home were included as sociodemographic factors. Chronotype was measured by a single item: “People distinguish between morning and evening persons, which type of person are you?” and categorized as a morning type (“obviously a morning person” or “more a morning person than an evening person”), an intermediate type (“neither a morning or evening person”), and an evening type (“more an evening person than a morning person”, or “obviously an evening person”). This single question has shown to be in excellent agreement with a quantitatively assessed chronotype based on sleep times in the Munich ChronoType Questionnaire (29). Self-perceived health was measured with a single item from the SF-36: “In general, would you say your health is…”, with a five-point answer scale ranging from poor to excellent (30) and categorized into poor/reasonable and good/very good/excellent.

### Statistical analysis

Percentages and means with corresponding standard deviations (SD) were used to describe the baseline characteristics of the study population. The daily work characteristics were presented as percentages and number of individuals and observations over the period of eight weeks.

Data on fatigue were linked to roster data on the same day, while data on sleep quality were linked to the roster data from the previous day as it assessed the last long sleep period that followed the previous day. Consequently, the number of observations for each shift characteristic slightly differed between fatigue and sleep quality.

Two complementary linear regression analyses, fixed- and random-effects models, were used to investigate associations between roster characteristics (type of shift, time between shifts, rotation direction, and number of successive night shifts) and fatigue and sleep quality. Both models accounted for repeated observations within individuals. The within- and between-person variance in the models with and without confounders were estimated to determine the impact of between-subject characteristics (covariates) on the proportion of model variance that is explained by within-person and between-person factors. Hausman tests were conducted to compare the random-effects and fixed-effects models for the relationships between roster characteristics, and fatigue and sleep quality. Under the null hypothesis, the random-effects model is preferred, whereas under the alternative hypothesis the fixed-effects model is the preferred model.

To determine whether time (day), location and department should be included as random intercept in the random-effects model, the differences in model fit using the AIC between models with and without these intercepts were estimated. Because time, location and department did not improve the model fit, they were not included as random intercepts. First, univariate analyses were performed to study the association between a specific roster characteristic, and fatigue or sleep quality. Second, multivariate random-effect models were conducted to examine the associations between roster characteristics and these outcomes, adjusted for age, gender, education, chronotype, general health, having children aged ≤12 years living at home, and type of shift. An association with P<0.05 was considered statistically significant.

For the two confounders (ie, chronotype and self-rated health) that showed the largest changes on the within- and between-variance, interaction terms between the confounder and type of shift (night shift versus other shifts) were added to the multivariate random-effects models. If statistically significant (P<0.10), the analyses were stratified. For reasons of privacy, cells with number of observations <20 are not presented.

Type of shift, time between shifts, rotation direction, and successive night shifts were included in the same way in the fixed effect models. As fixed-effects analyses use repeated measures with each person as his or her own control, time between shifts and rotation direction were only adjusted for type of shift. The statistical package R version 3.6.2 (package PLM) was used to conduct the random- and fixed-effects model analyses.

## Results

### Baseline characteristics

The study population consisted of 223 shift workers with a mean age of 47.4 years (SD 9.5) (table 1). The majority of the study population was male (53%) and intermediate educated (61%). A large majority mentioned their health as good or very good (81%) and 55% of the workers labelled themselves as an evening chronotype. The mean level of fatigue was 5.5 (SD 2.4) and the mean sleep quality was 6.4 (SD 2.4). The number of observations for each participant ranged from 7 to 56, with a median of 28 observations (interquartile range 14–39). Almost all workers had ≥1 night shift (96%; table 2). The number of observations differed for the outcomes fatigue and sleep quality to incomplete response to the daily EMA survey. For fatigue, 25% of the observations were night shifts, of which the majority was a single night shift (56%), followed by two (29%) and three in a row (9%). For the majority of the shifts, rest time was ≥16 hours (72%) and a stable direction of rotation (60%). A similar distribution of observations was obtained for sleep quality. Compared to all shift workers of the entertainment company, respondent were more often ≥45 years and performed more often front desk services.

**Table 1:** Baseline characteristics of the study population (N=223). [SD=standard deviation.]

	% (N)	Mean (SD)
Demographics		
Age (years)		47.4 (9.5)
Gender (male)	53 (117)	
Educational level		
Low	13 (30)	
Intermediate	61 (135)	
High	26 (58)	
Living with children aged <12 years	53 (119)	
Personal		
Chronotype		
Morning	19 (42)	
Intermediate	26 (58)	
Evening	55 (123)	
General self-rated health		
Poor/reasonable	18 (41)	
Good/ very good/ excellent	82 (182)	
Fatigue [range 0–10]		5.5 (2.4)
Sleep quality [range 0–10]		6.4 (2.2)

**Table 2:** Shift schedule characteristics. [SD=standard deviation]

	Persons	Daily responses for fatigue	Daily responses for sleep quality	Shift duration (hours)	Shift start time (hours)	Shift end time (hours)
					
	% (N)	% (N)	% (N)	Mean (SD)	Mean (SD)	Mean (SD)
Type of shift ^a^						
Morning	22 (48)	2 (109)	2 (100)	07:58 (01:05)	08:21 (01:28)	16:20 (01:44)
Day	82 (183)	14 (877)	14 (865)	07:37 (00:59)	12:00 (00:35)	19:37 (00:55)
Evening	63 (140)	6 (356)	6 (369)	07:04 (01:45)	15:23 (01:15)	22:27 (01:35)
Night	96 (215)	25 (1572)	27 (1656)	08:02 (00:51)	19:13 (01:31)	03:16 (01:10)
Day off following workday	99 (220)	21 (1280)	20 (1265)			
≥2 days off following a working day	98 (219)	32 (1979)	31 (1940)			
Successive night shifts						
1	96 (214)	56 (877)	58 (954)			
2	78 (173)	29 (453)	27 (448)			
3	37 (83)	9 (144)	10 (159)			
≥4	19 (43)	6 (97)	6 (94)			
Time between shfits (hours rest)						
<11 (quick return)	43 (95)	4 (114)	4 (108)			
11–16	84 (188)	24 (688)	23 (696)			
≥16	98 (219)	72 (2050)	73 (2181)			
Rotation direction						
Backward	83 (185)	19 (477)	21 (567)			
Stable	96 (214)	60 (1524)	60 (1605)			
Forward	84 (187)	22 (550)	19 (512)			

a Morning shift (starts after 06:00 and before 11:00 and mostly at 07:00 hours); day shift (starts after 11:00 and before 14:00; evening shift (starts after 14:00 and ends before 01:00 hours); night shift (starts after 14:00 and ends after 01:00 hours).

### Within- and between-subject variance

The results from the Hausman tests comparing random-effects and fixed-effects models for fatigue and sleep quality indicated that random-effects models were the preferred model for both outcomes. The between-person variance was 63% for fatigue and 66% for sleep quality, meaning that a higher variance existed between workers compared to changes over time within individuals. The within- and between-person variance did not change when adding confounders to the models, except for chronotype and self-rated health (data not shown).

### Roster characteristics, and fatigue and sleep quality

Table 3 shows that a night shift (β=0.22; 95% CI 0.05–0.39; multivariate model) was associated with more fatigue. A day of following a workday, often including an evening or a night shift, was associated with more fatigue (β=0.51; 95% CI 0.34–0.68) and ≥2 days off was associated with less fatigue (β= -0.46; 95% CI -0.62– -0.29). Rest time of 11–16 hours (β=0.43; 95% CI 0.26–0.61) and a quick return (β=1.94; 95% CI 1.57–2.31) were associated with more fatigue compared to a rest period of ≥16 hours. Compared to forward rotation, stable rotation (β=0.22; 95% CI 0.01–0.43) and backward rotation (β=0.49; 95% CI 0.23–0.74) were also associated with more fatigue. Two or more successive night shifts were associated with more fatigue (β=0.69; 95% CI 0.49–0.88) for two successive night shifts up to β of 0.58 (95% CI 0.16–0.99) for ≥4 successive night shifts. A single night shift was not associated with fatigue (β= -0.01; 95% CI -0.16–0.13).

**Table 3:** Random-effects model of the relationships between shift schedule characteristics with fatigue and sleep quality (scale 0 –10; higher score indicating more fatigue and poorer sleep quality). **Boldface indicates statistical significance (P<0.05)**.[CI=confidence interval.]

	Fatigue		Sleep quality	
	
	Univariate model ^a^	Multivariate model ^b^	Univariate model ^a^	Multivariate model ^b^
			
	B (95% CI)	B (95% CI)	B (95% CI)	B (95% CI)
Type of shift ^c^				
Morning	0.13 (-0.28–0.55)	0.13 (-0.28–0.54)	0.28 (-0.14–0.70)	0.28 (-0.14–0.70)
Day	Ref.	Ref.	Ref.	Ref.
Evening	-0.07 (-0.32–0.17)	-0.08 (-0.32–0.17)	0.10 (-0.14–0.34)	0.09 (-0.15–0.34)
Night shift	**0.22 (0.05–0.39)**	**0.22 (0.05–0.39)**	**0.64 (0.47–0.80)**	**0.64 (0.47–0.80)**
Day off following workday	**0.51 (0.34–0.68)**	**0.51 (0.34–0.68)**	0.10 (-0.07–0.27)	0.10 (-0.07–0.27)
≥2 days off following a working day	**-0.45 (-0.62– -0.29)**	**-0.46 (-0.62– -0.29)**	-0.06 (-0.22–0.10)	-0.06 (-0.22–0.10)
Time between shifts (hours rest)				
>16	Ref.	Ref.	Ref.	Ref.
11–16	**0.40 (0.23–0.57)**	**0.43 (0.26–0.61)**	-0.07 (-0.24–0.10)	0.01 (-0.18–0.16)
<11 (quick return)	**1.64 (1.28–2.00)**	**1.94 (1. 57–1.2.31)**	-0.28 (-0.64–0.10)	0.15 (-0.23–0.53)
Rotation direction				
Forward	Ref.	Ref.	Ref.	Ref.
Stable	**0.20 (-0.01–0.41)**	**0.22 (0.01–0.43)**	-0.16 (-0.35–0.02)	-0.04 (-0.23–0.14)
Backward	**0.33 (0.09–0.57)**	**0.49 (0.23–0.74)**	**-0.32 (-0.54– -0.08**)	0.12 (-0.13–0.37)
Successive night shifts				
0	Ref.	Ref.	Ref.	Ref.
1	-0.01 (-0.16–0.13)	-0.01 (-0.15–0.14)	**0.69 (0.55–0.82)**	**0.69 (0.55–0.82)**
2	**0.68 (0.49–0.87)**	**0.69 (0.49–0.88)**	**0.55 (0.36–0.74)**	**0.56 (0.37–0.74)**
3	**0.45 (0.12–0.79)**	**0.56 (0.13–0.79)**	**0.47 (0.16–0.78)**	**0.47 (0.16–0.78)**
≥4	**0.58 (0.16–0.99)**	**0.58 (0.16–0.99)**	0.26 (-0.15–0.67)	0.27 (-0.14–0.67)

a Unadjusted model per roster characteristic.

b Model per roster characteristic adjusted for age, gender, education, children living at home under the age of 12, general health, and chronotype. Time between shifts and rotation direction additionally adjusted for type of shift.

c Morning shift (starts after 06:00 and before 11:00 and mostly at 07:00 hours); day shift (starts after 11:00 and before 14:00 hours); evening shift (starts after 14:00 and ends before 01:00 hours); night shift (starts after 14:00 and ends after 01:00 hours).

Regarding sleep quality, a night shift was associated with poorer sleep quality (β=0.64; 95% CI 0.47–0.80); table 3). Successive night shifts were associated with a poorer sleep quality. The β decreased with more nights shifts as the β ranged from 0.69 (95% CI 0.55–0.82) for a single night shift to 0.27 (95% CI -0.14–0.67) for ≥4 night shifts. Rotation direction and time between shifts were no longer associated with sleep quality after adjusting for confounders.

The results of the fixed-effects analyses showed comparable results (supplementary table S1, www.sjweh.fi/article/3964).

### Chronotype and self-rated health as moderators

The effects between some roster characteristics and sleep quality and fatigue were moderated by chronotype and self-perceived health status. Individuals with a morning or intermediate chronotype working in a night shift had a poorer sleep quality compared to those with an evening chronotype. Moreover, workers with an intermediate chronotype with ≥1 successive night shifts had a poorer sleep quality than workers with an evening chronotype (table 4). Regarding self-perceived health, the effects of night shift and more successive night shifts on sleep quality and fatigue was larger for workers with a poor self-perceived health compared to those with a good self-perceived health (table 5). The effects of time between shifts on fatigue were also larger for workers with poor health.

**Table 4:** Random-effects model of the relationships between shift schedule characteristics and sleep quality, stratified by morning and evening chronotype (scale 0–10; higher score indicating poorer sleep quality). **Boldface indicates statistical significance (P< 0.05)**. [CI=confidence interval.]

	Morning chronotype	Intermediate chronotype	Evening chronotype
		
	B (95% CI)^a^	B (95% CI) ^a^	B (95% CI) ^a^
Type of shift ^b^			
Morning	0.27 (-0.62–1.17)	0.31 (-0.39–1.01)	0.26 (-0.38–0.90)
Day	Ref.	Ref.	Ref.
Evening	0.35 (-0.16–0.86)	0.06 (-0.40–0.52)	0.01 (-0.33–0.35)
Night	**1.14 (0.74–1.55)**	**0.66 (0.37–0.96)**	**0.48 (0.25–0.71)**
Day off following workday	**0.43 (0.03–0.83**)	-0.10 (-0.41–0.20)	0.07 (-0.16–0.31)
≥2 days off following a working day	-0.09 (-0.46–0.29)	-0.05 (-0.34–0.24)	-0.06 (-0.29–0.17)
Time between shifts (hours rest)			
>16	Ref.	Ref.	Ref.
11–16	-0.15 (-0.60–0.29)	**0.42 (0.11–0.77)**	-0.15 (-0.37–0.06)
<11 (quick return)	-0.07 (-1.14–1.00)	-0.20 (-0.73–0.48)	0.41 (-0.12–0.95)
Rotation direction			
Forward	Ref.	Ref.	Ref.
Stable	-0.32 (-0.80–0.16)	0.21 (-0.13–0.55)	-0.08 (-0.33–0.18)
Backward	-0.12 (-0.73–0.49)	0.40 (-0.04–0.85)	0.06 (-0.29–0.40)
Successive night shifts			
0	Ref.	Ref.	Ref.
1	**1.22 (0.90–1.55)**	**0.56 (0.31–0.80)**	**0.59 (0.41–0.77)**
2	**0.66 (0.14–1.18)**	**0.89 (0.55–1.23)**	**0.36 (0.11–0.61)**
3	..^c^	**1.15 (0.47–1.83)**	0.31 (-0.06–0.68)
≥4	..	..	0.07 (-0.40–0.54)

a Analyses model per roster characteristic adjusted for age, gender, education, children living at home under the age of 12, general health, and type of shift. Successive night shifts was not adjusted for type of shift.

b Morning shift (starts after 06:00 and before 11:00 and mostly at 07:00 hours); day shift (starts after 11:00 and before 14:00 hours); evening shift (starts after 14:00 and ends before 01:00 hours); night shift (starts after 14:00 and ends after 01:00 hours).

c Censored due to number of observations is too low (<20) for reliable estimations.

**Table 5:** Random-effects model of the relationships between shift schedule characteristics with fatigue and sleep quality, stratified by health status (scale 0 to 10; higher score indicating poorer sleep quality). **Boldface indicates statistical significance (P< 0.05).** [CI=confidence interval.]

	Fatigue		Sleep quality	
	
	Poor Health B (95% CI) ^a^	Good health B (95% CI) ^a^	Poor Health B (95% CI) ^a^	Good health B (95% CI) ^a^
Type of shift ^b^				
Morning	0.30 (-0.61–1.20)	0.08 (-0.38–0.55)	0.30 (-0.58–1.18)	0.29 (-0.19–0.77)
Day	Ref.	Ref.	Ref.	Ref.
Evening	0.12 (-0.50–0.64)	-0.12 (-0.39–0.15)	0.28 (-0.32–0.88)	0.06 (-0.20–0.33)
Night shift	**0.38 (-0.06–0.81)**	**0.20 (0.01–0.38)**	**0.99 (0.59–1.39)**	**0.58 (0.40–0.76)**
Day off following workday	**0.68 (0.27–1.09)**	**0.46 (0.28–0.65)**	0.11 (-0.29–0.50)	0.10 (-0.08–0.29)
≥2 days off following a working day	**-0.64 (-1.02– -0.26)**	**-0.40 (-0.58– -0.22)**	-0.20 (-0.57–0.16)	-0.00 (-0.19–0.17)
Time between shifts (hours rest)				
>16	Ref.	Ref.	Ref.	Ref.
11–16	**0.21 (0.21–1.15)**	**0.38 (0.20–0.57)**	-0.16 (-0.63–0.31)	0.01 (-0.17–0.18)
<11 (quick return)	**1.41 (1.41–3.54)**	**1.84 (1.45–2.23)**	0.60 (-0.01–1.20)	-0.04 (-0.32–0.23)
Rotation direction				
Forward	Ref.	Ref.	Ref.	Ref.
Stable	0.19 (-0.36–0.74)	0.18 (-0.06–0.42)	-0.07 (-0.56–0.42)	-0.06 (-0.26–0.15)
Backward	0.53 (-0.11–1.16)	0.03 (-0.27–0.32)	0.60 (-0.01–1.20)	-0.04 (-0.32–0.23)
Successive night shifts				
0	Ref.	Ref.	Ref.	Ref.
1	0.31 (-0.07–0.69)	0.22 (-0.001–0.45)	**1.20 (0.86–1.53)**	**0.57 (0.43–0.72)**
2	**0.69 (0.13–1.24)**	**1.02 (0.76–1.28)**	**0.84 (0.34–1.33)**	**0.50 (0.29–0.70)**
3	0.83 (-0.09–1.75)	**0.73 (0.34–1.11)**	..	**0.45 (0.12–0.77)**
≥4	..^c^	**1.06 (0.59–1.54)**	..	0.36 (-0.07–0.80)

a Analyses model per roster characteristics adjusted for age, gender, education, children living at home under the age of 12, chronotype, and type of shift. Successive night shifts was not adjusted for type of shift.

b Morning shift (starts after 06:00 and before 11:00 and mostly at 07:00 hours); day shift (starts after 11:00 and before 14:00 hours; evening shift (starts after 14:00 and ends before 01:00 hours); night shift (starts after 14:00 and ends after 01:00 hours).

c Are censored due to number of observations is too low (<20) for reliable estimations.

## Discussion

Night shifts were related with more fatigue and poorer sleep quality among shift workers in the entertainment industry compared to day shifts or a day off. With more successive night shifts sleep quality improved yet remained relatively poor. The effect of night shifts on fatigue was not present at the first night shift but ≥2 successive night shifts were related to more fatigue. A quick return and 11–16 hours between shifts were associated with more fatigue compared to a rest of ≥16 hours. Stable and backward rotation were associated with more fatigue compared to forward rotation. No associations were found between time between shifts and rotation direction, and sleep quality. Workers with a morning or intermediate chronotype had a poorer sleep quality after a night shift than workers with an evening chronotype. This was also the case for workers with poorer health, and they also reported more fatigue than workers with night shifts and good health.

With regard to fatigue, previous studies using administrative working patterns data predominantly showed a higher risk for chronic fatigue after a night shift and quick returns (20, 23, 31). The current study contributed to the existing knowledge by providing insight in acute effects of additional roster characteristics such as rotation direction and number of night shifts on fatigue at the end of the day or shift. Whereas consensus exists that quick returns should be avoided (18, 21), evidence considering rotation direction and fatigue is limited as previous longitudinal studies mostly compared different shift work schedules which do not provide insight into the effects of specific roster characteristics (18). Forward rotation is supposed to be better for sleep and fatigue than backward rotation as it allows for a quicker adaptation of the circadian clock and implies more time off between shifts (18). Quick returns are an extreme example of a backward rotation with limited time to recover, probably explaining the larger effect on fatigue of quick returns than of backward rotation.

Regarding sleep quality, roster characteristics were less consistent associated with poorer sleep quality. First, in contrast with previous studies that reported none to limited adjustment of the biological clock and sleep quality with successive night shifts (25, 32, 33), the effects of night shifts on sleep quality decreased in the present study with more successive night shifts. An explanation of the contrasting results might be that next to the number of successive night shifts, the sequence also matters. Sleep quality was found to be worse for the last night shift irrespective of the number of successive night shifts (25). Surprisingly, also no effects were found between quick returns and rotation direction on sleep quality. Previous studies showed clear associations between backward rotation and quick return on sleepiness or sleep duration (21, 34). Nevertheless, among the shift workers, the sustained poor sleep quality may lead to accumulated sleep debt, which in turn may lead to fatigue and other health problems (10, 17). This is illustrated by our findings of increased fatigue after ≥2 night shifts and not after a single night shift. Increased levels of fatigue pertained on the first day off after a working day, often an evening or a night shift. Workers may suffer from partial sleep deprivation on their first day off leading to increased levels of fatigue at the end of the day. They seem to recover from this fatigue on their second day off. Overall, the current study showed that specific roster characteristics – limited time between shifts (in particular quick returns) and stable or backward rotation – negatively affects sleep problems followed by fatigue (having lower level of energy). In line with the discussion paper by Garde et al (18), optimization of work schedules, ie, meaning more time to recover, forward rotation and potentially less consecutive night shifts are probably helpful to combat the short-term effects but also the long-term health consequences of night work (35–37).

The current study showed that workers with morning or intermediate chronotype reported a poorer sleep quality after a night shift as well as on the first day after a working day, while the association between night work and fatigue was not moderated by chronotype. Previous research also indicated that sleeping after night shifts is curtailed for morning and intermediate chronotypes (9, 38) and this might have an impact on the sleep quality as well. This is supported by the study of Juda et al (39) that showed reduced sleep duration as well as poorer sleep quality in shift workers with earlier chronotype. More night shifts had in the present study a negative impact on sleep quality among intermediate chronotypes, and in contrast to expectations a positive effect among evening chronotypes. Evening types might adjust their sleep patterns over the course of successive night shifts, which reduces the impact on sleep issues. Shift workers with poor self-perceived health slept less well and were more fatigued after (a series) of demanding shifts [ie, (successive) night shift, quick returns and 11–16 hours between shifts]. This indicates that shift workers with poor health need more time to recover from night work, and that their shift schedule should include more time off work after night shifts compared to workers with good health. Although age had little effect on the reported results, both chronotype and health are associated with age. With increasing age, people become earlier chronotypes (40) and health problems become more prevalent (41). Considering an ageing shift worker population and suggested lower employability of older shift workers (42), optimizing the shift schedules to the needs of individual workers with particular attention to older workers is required in order to sustain a healthy and productive shift work population.

In this study self-rostering was available for the vast majority of the study population, which will introduce coping strategies to alleviate adverse effects of shift work on sleep quality and fatigue. It may be hypothesized that workers who cope well with many successive night shifts, backward rotation and quick returns will choose their shifts accordingly. Larger differences in effects of roster characteristics on sleep and fatigue according to their chronotype and health status may therefore be expected in shift workers in a collective and regular shift system, in which shifts are evenly distributed across workers and all rosters have the same pattern.

A major strength of this study is the study design which combines subjective data on fatigue and sleep quality linked to a register-based assessment of working patterns, which results in a large number of observations. In other shift work studies with daily measurements (eg, diary studies) usually 20–50 shift workers were followed for a period of 2–4 weeks (34, 43, 44), compared to >200 shift workers for a period of 8 weeks in this study. These studies usually have more extensive measurements, eg, a higher sampling rate to study the course of fatigue over the day, whereas our study focused on the exhaustion dimension of fatigue. To receive a high compliance toward the daily measurements and thereby a sufficient sample size to ensure variation in roster characteristics, the daily survey was short. This limited the opportunity to measure fatigue multiple times a day and other relevant variables like sleep duration, use of medication, workload or other stressful events that may impact sleep quality and fatigue. A limitation of our study is the specific study sample within the understudied entertainment industry where night shifts ended in 12 of the 14 locations at 04:00 hours at the latest. Sleepiness normally peaks after 04:00 hours and sleep duration is on average 45 minutes shorter compared to night shifts ending at 06:00 hours (45–47). Lower fatigue levels and longer and better sleep may therefore be expected in night shifts ending before 04:00 hours, thereby possibly underestimating the effects of night work compared to other populations working “full” night shifts. Next to the likely possibility of a relatively healthy study population due to the healthy (shift) worker effect (48), the study also consisted of a selective group of the initial group of workers who participated in the baseline survey (N=869; 26%). The demographics of the respondents were similar to all shift workers employed by the entertainment company, except that older and front desk workers were slightly overrepresented. Furthermore, evening chronotypes were highly prevalent in our study population, reflecting potential self-selection of evening chronotypes into this company. The moderation analyses on chronotype showed larger effects on sleep quality among morning chronotypes compared to evening chronotypes. This indicates that the self-selection process into these kind of jobs with primarily evening and night shifts may reduce the adverse effects of these shifts on sleep quality.

In conclusion, night shifts have a negative impact on fatigue and sleep quality of shift workers in the entertainment industry. Specific roster characteristics including quick returns and backward rotation and more consecutive nights increased fatigue but had no negative effect on sleep quality. Workers with an early and intermediate chronotype or workers with a poor self-perceived health reported lower sleep quality during night shifts, and those with a poor health also reported more fatigue. Optimizing roster characteristics is needed to ensure more time to recovery, less backward rotation, and potentially less consecutive night shifts, especially among the most vulnerable groups.

## Supplementary material

Supplementary material
