# Supplementary material for: The acute effects of working time patterns on fatigue and sleep quality using daily measurements of 6195 observations among 223 shift workers
Source: Scand J Work Environ Health. 2021 Aug 31;47(6):446–55. doi: 10.5271/sjweh.3964 (PMC8504543; doi:10.5271/sjweh.3964)
Supplement: Supplementary material [file SJWEH-47-446-S001.pdf]

The acute effects of working time patterns on fatigue and sleep quality using daily measurements of 6195 observations among 223 shift workers[1]

by Hardy A van de Ven, PhD,[2] Gerben Hulsegge, PhD, Thijmen Zoomer, MSc, Elsbeth M de Korte, MSc, Alex Burdorf, PhD, Karen M Oude Hengel, PhD

1. *Supplementary material*
2. *Correspondence to: Hardy van de Ven, Netherlands Organisation for Applied Scientific Research TNO, Schipholweg 77, 2316 ZL, Leiden, The Netherlands. [E-mail: hardy.vandeven@tno.nl]*

Table S1. Fixed effects model of the relationships between shift schedule characteristics and fatigue and sleep quality (scale 0 to 10; higher score indicating more fatigue and poorer sleep quality).

	Fatigue		Sleep quality	
	Unadjusted model	Adjusted type of shift	Unadjusted model	Adjusted type of shift
	B (95% CI)	B (95% CI)	B (95% CI)	B (95% CI)
Type of shift				
Morning shift	0.11 (-0.30–0.53)	NA	0.28 (-0.14–0.71)	NA
Day shift	Ref.	NA	Ref.	NA
Evening shift	-0.09 (-0.34–0.16)	NA	0.08 (-0.16–0.32)	NA
Night shift	0.22 (0.05–0.39)	NA	0.64 (0.47–0.81)	NA
Day off following workday	0.50 (0.33–0.68)	NA	0.10 (-0.07–0.27)	NA
Second or more days off following a working day	-0.46 (-0.63– -0.30)	NA	-0.07 (-0.24–0.09)	NA
Time between shifts				
>16h rest	Ref.	Ref.	Ref.	Ref.
11-16h rest	0.41 (0.23–0.58)	0.44 (0.26–0.61)	-0.09 (-0.26–0.09)	-0.03 (-0.20–0.13)
<11h rest quick return	1.65 (0.29–2.01)	1.94 (1.57–2.31)	-0.27 (-0.64–0.11)	0.15 (-0.23–0.54)
Rotation direction				
Forward	Ref.	Ref.	Ref.	Ref.
Stable	0.21 (0.003–0.42)	0.24 (0.03–0.45)	-0.13 (-0.32–0.06)	-0.01 (-0.20–0.18)
Backward	0.33 (0.09–0.57)	0.50 (0.24–0.75)	-0.32 (-0.55– -0.09)	0.13 (-0.13–0.38)

Successive night shifts				
No night shift	Ref.	Ref.	Ref.	Ref.
1 in a row	-0.003 (-0.15–0.14)	NA	0.69 (0.56–0.83)	NA
2 in a row	0.69 (0.50–0.89)	NA	0.57 (0.38–0.76)	NA
3 in a row	0.46 (0.13–0.80)	NA	0.48 (0.17–0.79)	NA
≥4 in a row	0.60 (0.18–1.02)	NA	0.31 (-0.10–0.72)	NA

Boldface indicates statistical significance ($p < 0.05$). NA: not applicable as it was not applicable to those analyses for type of shift.
